# Mid-Epidemic Forecasts of COVID-19 Cases and Deaths: A Bivariate Model Applied to the UK

**DOI:** 10.1155/2021/8847116

**Published:** 2021-02-12

**Authors:** Peter Congdon

**Affiliations:** School of Geography, Queen Mary University of London, Mile End Road, London E1 4NS, UK

## Abstract

**Background:**

The evolution of the COVID-19 epidemic has been accompanied by efforts to provide comparable international data on new cases and deaths. There is also accumulating evidence on the epidemiological parameters underlying COVID-19. Hence, there is potential for epidemic models providing mid-term forecasts of the epidemic trajectory using such information. The effectiveness of lockdown or lockdown relaxation can also be assessed by modelling later epidemic stages, possibly using a multiphase epidemic model.

**Methods:**

Commonly applied methods to analyse epidemic trajectories or make forecasts include phenomenological growth models (e.g., the Richards family of densities) and variants of the susceptible-infected-recovered (SIR) compartment model. Here, we focus on a practical forecasting approach, applied to interim UK COVID data, using a bivariate Reynolds model (for cases and deaths), with implementation based on Bayesian inference. We show the utility of informative priors in developing and estimating the model and compare error densities (Poisson-gamma, Poisson-lognormal, and Poisson-log-Student) for overdispersed data on new cases and deaths. We use cross validation to assess medium-term forecasts. We also consider the longer-term postlockdown epidemic profile to assess epidemic containment, using a two-phase model.

**Results:**

Fit to interim mid-epidemic data show better fit to training data and better cross-validation performance for a Poisson-log-Student model. Estimation of longer-term epidemic data after lockdown relaxation, characterised by protracted slow downturn and then upturn in cases, casts doubt on effective containment.

**Conclusions:**

Many applications of phenomenological models have been to complete epidemics. However, evaluation of such models based simply on their fit to observed data may give only a partial picture, and cross validation against actual trends is also valuable. Similarly, it may be preferable to model incidence rather than cumulative data, although this raises questions about suitable error densities for modelling often erratic fluctuations. Hence, there may be utility in evaluating alternative error assumptions.

## 1. Introduction

Epidemic forecasts have been an essential element in policy decisions regarding the COVID-19 epidemic, such as lockdown imposition and relaxation. Forecasting has been assisted by well-organized efforts to provide international data on new cases and deaths. These include the daily updated comparative data provided by the European Centre for Disease Prevention and Control (ECDC) (https://www.ecdc.europa.eu/en/publications-data), and monitoring profiles provided by John Hopkins University [1]. There is also a growing literature providing evidence on the parameters of the COVID-19 infection (for example, case fatality ratios and serial intervals). Hence, the potential occurs for epidemic models that are applicable to routinely collected data, which make use of accumulated evidence, and can provide forecasts for epidemics observed at mid-stage. Policy decisions in many countries (imposition of lockdowns, and later, relaxation) have been made based on trends in observed numbers of cases and deaths, while admitting these may be subject to measurement error, for example, identified cases may understate total numbers infected; there are fluctuations in daily new cases due to variation in daily testing, and there may be COVID-19 diagnostic errors.

Here, we focus on a practical forecasting approach using routinely available data on new cases and deaths (from ECDC) to estimate parameters in a bivariate version of the Reynolds phenomenological model. Implementation is based on Bayesian inference principles, incorporating accumulated evidence on relevant parameters via informative priors. The operation and utility of this approach is demonstrated using data on new cases and deaths in the United Kingdom (UK), with a focus on predictive accuracy for 20-day-ahead forecasts of cases and deaths, based on mid-epidemic data. Other policy relevant parameters such as the effective reproduction ratio and case fatality ratio are also estimated in analysis of longer-term epidemic data.

The use of a bivariate approach provides originality compared to existing research, which, in the case of phenomenological models, is limited to analysing incidence only. Some studies have mentioned how the mortality curve parallels the epidemic curve [2], and we formalize these linkages under a bivariate approach. The benefits of a bivariate approach include the ability to monitor and forecast severity measures such as the case fatality ratio. We also consider issues in modelling daily incidence and new deaths, as opposed to cumulative incidence and mortality. There are methodological issues in analysing cumulative outcomes, discussed below, but also questions (not so far considered in the literature) on how best to represent the overdispersion present in uncumulated outcomes. The analysis below provides new evidence on the relative fit and forecasting performance of different ways of representing Poisson overdispersion and shows that the usually adopted negative binomial performs less well than other options.

The implications of the research are that effective medium-term forecasts of COVID-19 incidence and mortality can be provided by the proposed methodology. Such forecasts are useful in planning healthcare provision and assessing closeness to full capacity in hospital bed occupancy [3, 4]. At the time of writing, daily COVID-19 hospitalisation data were not available for the UK. However, extension of the bivariate approach to include incidence, mortality, and hospitalisations provides additional scope for forecasting of indicators relevant to severity assessment [5, 6]. Another possible outcome in a multivariate model is recovered cases (cf. [4]), with a focus then on the ratio of predicted recovered to predicted confirmed cases as an indicator of care need and effectiveness of interventions. The methodology also presents a way to monitor longer-term infection numbers leading to early detection of incipient upturns in infection numbers, via continuous monitoring and forecasting of the effective reproduction ratio.

In the following sections, we review relevant literature and research gaps (Section 2) and set out the methodology (Section 3). We then consider aspects of the UK case study application (Section 4), present the results (Section 5), and discuss the implications of the study's findings and methodology in the context of broader research (Section 6).  Section 7 provides concluding remarks.

## 2. Related Research and Research Gaps

Commonly applied approaches to quantitative modelling of aggregate epidemic data differ in the data inputs they require, their assumptions, their estimability, and their scope for practical application to making forecasts. Commonly applied methods include phenomenological growth models [7], such as the Richards family of densities [8], and variants of the susceptible-infected-recovered (SIR) compartment model [9]. Phenomenological models are parameterised in terms of epidemic trajectories and provide estimates of crucial epidemiological parameters [10, 11], while avoiding the complexity of more formal mechanistic models of disease transmission, which can be difficult to estimate and provide forecasts, and may not be realistic approximations to real epidemic dynamics [2, 9, 12].

As mentioned by Chowell et al. [13], phenomenological models are “particularly suitable when significant uncertainty clouds the epidemiology of an infectious disease.” By contrast, as noted in [14], compartmental transmission models may be based on untested assumptions such as random mixing between all individuals in a given population, may be sensitive to starting assumptions, and may provide estimates that differ considerably between models. Such models often rely on preset parameters, which may mean prediction uncertainty is understated. They may also be complex to specify when an epidemic has more than one phase, whereas multiphase phenomenological models [15] are available.

Regarding forecasts, the study by Zhao et al. [10] exemplifies application of phenomenological models to forecasts of the Zika epidemic in 2015. Autoregressive modelling of new cases, with potential for short-term forecasting, is illustrated (for foot and mouth disease) by the first-order autoregressive model of Lawson et al. [16], while (for multiple spatial units) the model of Bracher and Held [17] specifies a first-order autoregression based on the mean incidence in adjacent areas.

Regarding the COVID-19 epidemic in particular, studies with differing methodologies have been made, some of which forecast different aspects of the COVID epidemic or related health care need. In its impact on policy making in the UK and US, perhaps most influential has been the Imperial College model [18]. This is based on microsimulation with transmission through contacts between susceptible and infectious individuals in various settings, or randomly in the community, depending on spatial distance between contacts. A number of epidemic parameters (e.g., incubation periods and basic reproduction numbers) are preset. Forecasts are provided for deaths and hospital beds. Also, providing forecasts across countries is the model of the Institute for Health Metrics and Evaluation [14]. This has no underlying representation of epidemic dynamics, but is based on fitting a hierarchical parametric model for observed cumulative death rates in different countries, and then projecting these forward.

Various types of time series forecast of the COVID-19 epidemic have also been made, using ARIMA models [4, 19, 20], exponential smoothing [21], or autoregression in cases; for example, the study by Johndron et al [22] postulates daily deaths as a lagged function of earlier new cases. Applications of phenomenological models to COVID-19 incidence forecasts include the studies in [23, 24].

One may identify some research gaps in the existing literature. Thus, existing applications (e.g., [23] in the case of COVID-19) have most commonly been to incidence data and have not considered interplay between outcomes in terms of a bivariate model. However, examples of potentially interlinked bivariate outcomes from COVID-19, and other epidemics, include incidence and mortality, and incidence and hospitalisations. The study in [25] converts COVID-19 incidence, mortality, and case fatality into beta variables and applies univariate beta regression models to each outcome. The study in [4] applies separate ARIMA models to incidence and recovered cases. However, separate univariate regressions or time series models do not reflect potential interlinkages between the processes that facilitate the setting of model assumptions or, in the case of Bayesian analysis, facilitate the setting of priors on model parameters. Regarding forecasting, wider experience of time series modelling (with nonepidemic applications) shows the benefit of borrowing strength over outcomes [26].

Furthermore, many existing applications, such as studies in [11, 25] in the case of COVID-19, study in [27] in the case of H1N1 and Ebola, and study in [10] in the case of Zika, have been to cumulative incidence. However, the drawbacks of studying cumulative incidence have been pointed out [28, 29]. Cumulative incidence data have serially correlated measurement error, leading to understatement of parameter uncertainty. As stated in [29], “independence of sequential measurement errors,…is clearly violated when observations are accumulated through time.” However, estimation using new cases or deaths (uncumulated) puts a much greater focus on how to deal with stochastic variation in the data. For daily data, fluctuations in new events may be considerable (including daily “spikes”), whereas cumulative cases and deaths are usually smooth functions. There are choices in how to model the overdispersion in new events, based on Poisson mixtures [30], but in the epidemic literature, use of the negative binomial is standard, and no evaluations of its relative performance are available in the literature so far.

## 3. Methods

### 3.1. Phenomenological Models

Basic phenomenological models for epidemic trajectories include the logistic, Gompertz, and Rosenzweig, which have been the basis for a range of generalisations [7, 8]. Application of the logistic model to COVID-19 is exemplified by the studies of Batista [31] and Shen [32]. For time *t*, the logistic model for new cases *C*′(*t*) and cumulative cases *C*(*t*) is

(1) $$\begin{array}{c} C^{\prime }\left(t\right)=rC\left(t\right)\left[1-\frac{C\left(t\right)}{K}\right],\\ C\left(t\right)=\frac{K}{\left[1+e^{-r\left(t-\tau_{L}\right)}\right]}, \end{array}$$

where *K* is the maximum number of cases (final epidemic size), *r* > 0 measures the intensity of exponential growth in cases in the early epidemic phase, and *τ*_*L*_ is the inflection point where new cases are highest. The Richards model [33] modifies the logistic incidence function to

(2) $$\begin{array}{c} C^{\prime }\left(t\right)=rC\left(t\right)\left[1-{\left(\frac{C\left(t\right)}{K}\right)}^{a}\right], \end{array}$$

with solution

(3) $$\begin{array}{c} C\left(t\right)=\frac{K}{{\left[1+e^{-r\left(t-\tau \right)}\right]}^{1/a}}. \end{array}$$

The parameter *a* > 0 modifies the incidence decline phase of the logistic, that is, measures the extent of deviation from the standard logistic curve. The turning point *τ*, when incidence peaks, is obtained when *C*(*t*) equals *K*(1+*a*)^−1/*a*^ [34]. The peak incidence is important for the healthcare planning, for example, aligning the forecast peak with hospital bed capacity [35].

Other commonly used models are the Gompertz model [36] with

(4) $$\begin{array}{c} C^{\prime }\left(t\right)=rC\left(t\right)\log\left[\frac{K}{C\left(t\right)}\right], \end{array}$$

while the Rosenzweig model [27] has

(5) $$\begin{array}{c} C^{\prime }\left(t\right)=rC\left(t\right)\left[{\left(\frac{C\left(t\right)}{K}\right)}^{a}-1\right]. \end{array}$$

The incidence function represented by *C*′(*t*) can be used to define mean incidence in statistical likelihoods for new cases data. Thus, time series of incidence counts can often be satisfactorily modelled as a Poisson, with means defined by *C*′(*t*) functions [37, 38]. Similarly, the cumulative cases function *C*(*t*) can be used to define mean epidemic size in models for cumulative case counts [27, 39].

While for smaller epidemics, a Poisson density for mean incidence may be applied, for larger epidemics such as COVID-19, a negative binomial density is often preferred, both because of large incidence counts and to represent often erratic incidence fluctuations that lead to overdispersion relative to the Poisson [29]. However, the literature does not contain any evaluations of the negative binomial to represent overdispersion. There are a number of other overdispersed versions of the Poisson that can be achieved by mixing the Poisson with a suitable density (e.g., a lognormal density) [30, 40], and this may be beneficial in detecting unusual observations.

### 3.2. Model Specification: Poisson Overdispersion

Consider the Richards model parameters. Let *c*_*t*_ and *C*_*t*_ denote incidence and cumulative incidence counts at times *t*=1,…, *T* (days in the case of COVID data from ECDC). We condition on the first case or cases (i.e., the first observation) and take incidence at time *t* as a function of cumulative cases at *t* − 1, so that for a Poisson model for cases (with subscript *c* for parameters) we have

(6) $$\begin{array}{c} c_{t}∼\text{Poisson}\left(\mu_{ct}\right),\;\mu_{ct}=r_{c}C_{t-1}\left[1-{\left(\frac{C_{t-1}}{K_{c}}\right)}^{a_{c}}\right],\;t=2,\ldots ,T. \end{array}$$

In practice, many epidemic datasets are overdispersed relative to the Poisson, and epidemic studies generally adopt a negative binomial model instead. We can specify an overdispersed model (including the negative binomial) by introducing multiplicative random effects ([40], Equation (2)), such that the Poisson means for incidence are specified by

(7) $$\begin{array}{c} \mu_{ct}=r_{c}C_{t-1}\left[1-{\left(\frac{C_{t-1}}{K_{c}}\right)}^{a_{c}}\right]\epsilon_{ct}, \end{array}$$

where *ϵ*_*ct*_ are positive random effects. For the Poisson-gamma model (equivalent to a negative binomial), *ϵ*_*ct*_ are gamma distributed with mean 1, namely,

(8) $$\begin{array}{c} \epsilon_{ct}∼\text{Gamma}\left(\lambda_{c},\lambda_{c}\right), \end{array}$$

where 1/*λ*_*c*_ is the overdispersion parameter mentioned by [29]. Note that the assumed parameterisation of the gamma density with random variable *x* is

(9) $$\begin{array}{c} p\left(x|a,b\right)=\frac{b^{a}}{\Gamma \left(a\right)}x^{a-1}e^{-bx}. \end{array}$$

Other options in (7) are to take *u*_*ct*_=log(*ϵ*_*ct*_) as normally distributed [40]:

(10) $$\begin{array}{c} u_{ct}∼\text{Normal}\left(0,\sigma_{uc}^{2}\right), \end{array}$$

or Student *t* distributed [41],

(11) $$\begin{array}{c} u_{ct}∼\text{Student}-t\left(0,\sigma_{uc}^{2},\nu_{c}\right), \end{array}$$

where *ν*_*c*_ is a degrees-of-freedom parameter. These two options define the Poisson-lognormal (PLN) and Poisson-log-Student (PLS) options, respectively [30]. The PLN and PLS representations may provide a more robust alternative to the Poisson-gamma [40–44], as their tails are heavier than for the gamma distribution, and have been found to be better at accommodating outliers (such as daily “spikes” in an epidemic application).

### 3.3. A Joint Model for New Cases and New Deaths

In the analysis below, we apply a bivariate estimation with both new cases and deaths modelled using the Richards specification. Thus, denote *d*_*t*_ and *D*_*t*_ as new and cumulative deaths at time *t*. The joint likelihood for an overdispersed Poisson model for both outcomes then specifies

(12) $$\begin{array}{c} c_{t}∼\text{Poisson}\left(\mu_{ct}\right), \end{array}$$

(13) $$\begin{array}{c} \mu_{ct}=r_{c}C_{t-1}\left[1-{\left(\frac{C_{t-1}}{K_{c}}\right)}^{a_{c}}\right]\epsilon_{ct}, \end{array}$$

(14) $$\begin{array}{c} \mu_{dt}=r_{d}D_{t-1}\left[1-{\left(\frac{D_{t-1}}{K_{d}}\right)}^{a_{d}}\right]\epsilon_{dt},\;t=2,\ldots ,T. \end{array}$$

For a Bayesian application, we need to specify prior densities, or priors for short, for the parameters. For the epidemic size parameter *K*_*c*_, a diffuse prior confined to positive values, such as a diffuse gamma density, for example, Gamma(1, *ε*) or Gamma(*ε*, *ε*), with *ε* small, was found to lead to convergence problems. As noted in [31], “…in the early stage, the logistic curve follows an exponential growth curve, so the estimation of *K* is practically impossible.” This difficulty persists when an epidemic is past its peak but early in a downturn.

However, Batista [31] mentions a relationship (for the logistic model) between successive cumulative case counts that may assist in providing a prior for *K*_*c*_. Specifically, for three points spaced *m* time units apart, one may obtain the relationship for a point estimator of *K*_*c*_, namely,

(15) $$\begin{array}{c} K_{c}^{e}=\frac{C_{t-m}\left(C_{t-2m}C_{t-m}-2C_{t-2m}C_{t}+C_{t-m}C_{t}\right)}{\left(C_{t-m}^{2}-C_{t}C_{t-2m}\right)}. \end{array}$$

One may use this point estimator to define a prior mean for *K*_*c*_ in the Richards model (which is a generalisation of the logistic). Specifically, one may take a lognormal density prior for *K*_*c*_, with log(*K*_*c*_^*e*^) as mean, and a suitable variance, such that the prior is still relatively diffuse. For example, suppose *K*_*c*_^*e*^ is 250,000, and the variance in the lognormal is set at 1. Then, the 97.5 percentile for the lognormal prior is 1.77 million.

In the bivariate specification (for new cases and new deaths jointly), we seek to share prior information between outcomes. One option for the prior on *K*_*d*_ (the final death total) is as a function of *K*_*c*_, namely,

(16) $$\begin{array}{c} K_{d}=\Phi K_{c}, \end{array}$$

where Φ is a form of case fatality ratio (CFR). An informative prior for Φ could be based on the COVID experience in similar countries, or on experience of epidemics of similar diseases. Considering the first option, and an appropriate prior for analysing UK data, an informative prior for Φ could be provided by the case fatality ratio across the European Union (the UK being no longer an EU member). International information on case fatality is provided at https://ourworldindata.org/mortality-risk-covid#the-current-case-fatality-rate-of-covid-19.

Alternatively, one may link *K*_*c*_ and *K*_*d*_ using both the point estimator *K*_*c*_^*e*^ and a case fatality ratio; namely, *K*_*d*_^*e*^=Φ*K*_*c*_^*e*^. Then, a lognormal density prior can be taken for *K*_*d*_, with log(*K*_*d*_^*e*^)=log(*K*_*c*_^*e*^)+log(Φ) as the mean, and a suitable variance such that the prior is still relatively diffuse.

Another possible prior to link ultimate cases and deaths would involve a time series in time-specific case fatality ratios, such as an autoregression *ϕ*_*t*_ ~ *N*(*ρϕ*_*t*−1_, *σ*_*ϕ*_^2^), with *ϕ*_*t*_ estimated from cumulative data on deaths and cases. Some analyses of epidemics show that the CFR early in an epidemic may underestimate later values [45], in which case the prior on Φ may be constrained to exceed the final *ϕ*_*t*_ based on observed data. With regard to COVID-19, this pattern may not necessarily apply, with the US (for example) showing a decline in CFRs at later epidemic stages. There is also evidence that the mortality to infection ratio (a more precise measure than the CFR) has fallen [46]. To allow for such a scenario, the prior for Φ could be centred at the last observed *ϕ*_*t*_, rather than constrained to exceed it.

Joint priors on other parameters could be considered, for example, bivariate normal priors on the logs of *r*_*c*_ and *r*_*d*_, or on the logs of *a*_*c*_ and *a*_*d*_. In the empirical analysis below, we focus on priors linking the final epidemic and death total parameters, *K*_*c*_ and *K*_*d*_, as these are an important influence on forecasts.

### 3.4. Medium-Term Forecasts

Many applications of phenomenological models are to historic data on epidemics, where the epidemic has run to its full extent. Here, we consider applications to incomplete epidemics (e.g., epidemics observed to their mid-point or early in the downturn), and to forecasts using such data. Forecasts at an intermediate point within the observation span are of interest in themselves for policy purposes. However, they can also be used in comparative model evaluation by using cross validation, with only some data used for estimating the model, and some held out for validation.

Thus, suppose the training sample is formed by observations up to time *M* < *T*, while the *F* subsequent observations at times *t*=*M*+1,…, *M*+*F* (where *M*+*F* ≤ *T*) are used as a validation sample. Predictions *c*_new,*M*+1_ and *d*_new,*M*+1_ for new cases and deaths at time *M*+1 are based on observed cumulative counts *C*_*M*_ and *D*_*M*_. As usual in Bayesian inference, predictions are obtained as replicate data sampled from the posterior predictive densities *p*(*c*_new_*|Y*)=∫*p*(*c*_new_*|Y*, *θ*)d*θ* and *p*(*d*_new_*|Y*)=∫*p*(*d*_new_*|Y*, *θ*)d*θ*, where *Y*=(*c*, *d*) are data on new cases and deaths, and *θ* are parameters in the joint model of Section 3.3 [47].

Predicted cumulative counts at time *M*+1 are then obtained as *C*_new,*M*+1_=*C*_new,*M*_+*c*_new,*M*+1_ and *D*_new *M*+1_=*D*_new,*M*_+*d*_new,*M*+1_. Predicted new cases and deaths at *M*+2, namely, *c*_new,*M*+2_ and *d*_new *M*+2_, are then sampled from the appropriate phenomenological model form, based on *C*_new,*M*+1_ and *D*_new,*M*+1_. Cumulated cases and deaths at *M*+2 are then obtained by adding predicted new cases and deaths for *M*+2 to *C*_new,*M*+1_ and *D*_new,*M*+1_. This process is continued until time *M*+*F*.

Fit can be assessed by whether credible intervals for predictions in the cross-validation period include actual incidence and new deaths. Also relevant are probabilities of overprediction or underprediction. For example, consider predicted new cases *c*_new,*s*,*t*_ for the validation period *t*=*M*+1,…, *M*+*F*, and for MCMC samples *s*=1,…, *S*, and let average new predicted cases during the validation period (the average over *F* days) at iteration *s* be denoted ${\bar{c}}_{\text{new},s,M+1:M+F}$. We want to compare average predicted new cases with average observed new cases, ${\bar{c}}_{M+1:M+F}$, during the validation period. Probabilities of overprediction can be obtained from binary indicators:

(17) $$\begin{array}{c} O_{s}^{\left(c\right)}=I\left({\bar{c}}_{\text{new},s,M+1:M+F}>{\bar{c}}_{M+1:M+F}\right),\;s=1,\ldots ,S, \end{array}$$

where *I*(*A*)=1 if condition *A* is true, and *I*(*A*)=0 otherwise. Thus, at each iteration, we compare average new cases (modelled) during the validation period with actual average new cases.

Probabilities of overprediction for new cases, *ω*_*c*_, are estimated as ∑_*s*=1_^*S*^(*O*_*s*_^(*c*)^/*S*). Probabilities of underprediction can be obtained as 1 − *ω*_*c*_. A satisfactory prediction would have 0.05 < *ω*_*c*_ < 0.95, with *ω*_*c*_ over 0.95 indicating a high probability of overprediction, while *ω*_*c*_ under 0.05 indicates a high probability of underprediction. Underprediction means underforecasting of future cases and may lead to incorrect inferences regarding epidemic control, as it implies a lessening in incidence earlier than actually occurred.

### 3.5. Longer-Term Epidemic Monitoring, Effective Reproduction Ratios, and Case Fatality Ratios

Strategic decisions regarding containment of the COVID-19 epidemic, in the UK and other countries, have depended on trends in new infections and deaths, but also on the effective reproduction rate. Thus, in the UK, the choice on whether or not to relax the initial COVID lockdown restrictions was based on five criteria, with two being numeric: first, “a sustained and consistent fall in daily death rates” and second that the “rate of infection is decreasing to manageable levels,” meaning that the effective reproduction ratio is demonstrably below 1. The reproduction rate may also become especially relevant at later epidemic stages (postlockdown), after a downturn from the initial peak and after lockdown measures have been relaxed. Here, the concern is to prevent a resurgence of infection, indicated by an upturn in *R*_*t*_.

In the case of a protracted downturn, but with new cases still occurring, the concern is especially that there may be a substantial resurgence in cases, and possibly also deaths, at some point. This scenario is colloquially known as a “second wave,” and in most European countries, there have been pronounced second waves in the COVID-19 epidemic during 2020, albeit at different times. Such a resurgence indicates use of a multiphase model [15], with a second phenomenological model applied to data after a latent switch-point between epidemic regimes. Note that at the time of writing (August 2020), a fully developed second wave had not yet happened in the UK, although the signs were of an upturn in *R*_*t*_, as the analysis below confirms.

In planning for hospital care, longer-term trends in disease severity may be relevant. For example, an upturn in cases may reflect more cases among younger people at lower mortality risk. Hence, trends in, and forecasts of, the case fatality rate are an important aspect of strategic management. In a bivariate model of cases and deaths, as here, we can trace the modelled CFR through time, where the modelled CFR at day *t* is given by the ratio of predicted total deaths to predicted total cases *D*_new,*t*_/*C*_new,*t*_. In a natural extension of the bivariate model to include hospitalisations, trends in, and forecasts of, hospitalisation rates (ratios of hospitalised cases to total cases) can also be estimated.

Assume there is some evidence from new cases data of an upturn in cases, even before a second wave epidemic is fully established. Consider a model for new cases only. Then, a two-phase model to reflect the upturn would involve two phenomenological models, before and after a latent switch-point, with each model having distinct parameters. Denote the single switch-point as *κ*_*c*_, such that for a two-phase Richards model,

(18) $$\begin{array}{c} c_{t}∼\text{Poisson}\left(\mu_{ct}\right),\mu_{ct}=I\left(t<\kappa_{c}\right)r_{c1}C_{t-1}\left[1-{\left(\frac{C_{t-1}}{K_{c1}}\right)}^{a_{c1}}\right]+I\left(t\geq \kappa_{c}\right)r_{c2}C_{t-1}\left[1-{\left(\frac{C_{t-1}}{K_{c2}}\right)}^{a_{c2}}\right], \end{array}$$

where *I*(*A*)=1 when condition *A* is true, and 0 otherwise. Parameters, such as (*r*_*c*1_, *r*_*c*2_), are differentiated by outcome and by wave. The parameter *κ*_*c*_ can be assigned a uniform prior (on a positive interval) or a positive valued prior, such as an exponential density. If there is a second wave upturn in deaths also, then a bivariate model can be used, with switch-points *κ*_*d*_ assumed later for deaths than cases, due to possible delays in mortality upturns following incidence upturns. A three-phase model for cases would have two switch-points, with mean

(19) $$\begin{array}{c} \mu_{ct}=I\left(t<\kappa_{c1}\right)r_{c1}C_{t-1}\left[1-{\left(\frac{C_{t-1}}{K_{c1}}\right)}^{a_{c1}}\right]+I\left(\kappa_{c2}>t\geq \kappa_{c1}\right)r_{c2}C_{t-1}\left[1-{\left(\frac{C_{t-1}}{K_{c2}}\right)}^{a_{c2}}\right]\\ +I\left(t\geq \kappa_{c2}\right)r_{c3}C_{t-1}\left[1-{\left(\frac{C_{t-1}}{K_{c3}}\right)}^{a_{c3}}\right]. \end{array}$$

An estimator of the effective reproduction rate *R*_*t*_ at time *t* is based on predictions from this phenomenological model and from an estimate of the serial interval density. The serial interval is the time between symptom onset in an infected subject and symptom onset in the infectee. The serial interval density can be discretised in the form of weights *ρ*_*j*_, applied to serial interval lengths (in days) up to a maximum *J*. These can be used to estimate effective reproduction ratios *R*_*t*_ within a phenomenological model to analyse new cases; see the papers [37, 48, 49]. Thus, *R*_*t*_ can be estimated as

(20) $$\begin{array}{c} R_{t}=\frac{c_{\text{new},t}}{\sum_{j=0}^{J}\rho_{j}c_{\text{new},t-j}}, \end{array}$$

where *c*_new,*t*_ are predicted new case data from the phenomenological model. By virtue of the MCMC sampling strategy used below, we can readily obtain 95% credible intervals for *R*_*t*_, and the probability that *R*_*t*_ < 1, which is important for assessing epidemic containment strategy.

## 4. Model Application

**Figure 1:**
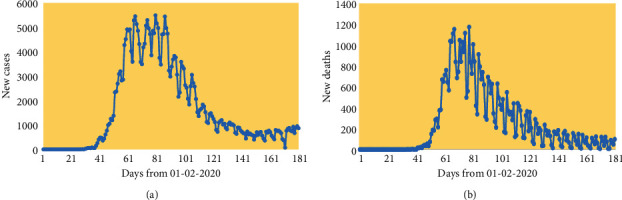
(a) Trends in daily new cases, UK; (b) trends in daily new deaths, UK.

**Figure 2:**
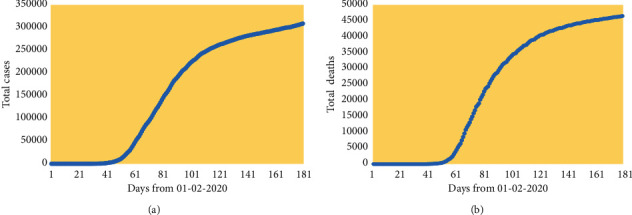
(a) Trends in total cases, UK; (b) trends in total deaths, UK.

We consider the application of the above methods to UK data on new cases and deaths from 1st February 2020 (when the first two cases of COVID-19 in the UK were reported according to ECDC). Observations are assigned dates as in the ECDC data, with times *t* in days from 1st February 2020. Figures 1(a) and 1(b) show daily trends in these outcomes up to 8th August 2020, with erratic fluctuations apparent in both outcomes. However, there is a broad downward trend in both outcomes from days 70 to 80, although with a more protracted decline as opposed to the steep initial upturn. Figures 2(a) and 2(b) show the relatively smooth evolution of cumulative cases and deaths.

### 4.1. Medium-Term Forecasts

For medium-term forecasts, we focus on the Richards bivariate model (Section 3.3) and compare three alternative error assumptions as discussed in Section 3.2: Poisson-gamma (PG), Poisson-lognormal (PLN), and Poisson-log-Student (PLS). The Student density is represented by a scale mixture of normals [50, 51]. Thus, for new cases, instead of taking

(21) $$\begin{array}{c} u_{ct}∼t\left(0,\sigma_{uc}^{2},\nu_{c}\right), \end{array}$$

where *ν*_*c*_ is the degree of freedom parameter and *u*_*ct*_=log(*ϵ*_*ct*_), we take

(22) $$\begin{array}{c} \delta_{ct}∼\text{Gamma}\left(\frac{\nu_{c}}{2},\frac{\nu_{c}}{2}\right),\\ u_{ct}∼N\left(0,\frac{\sigma_{uc}^{2}}{\delta_{ct}}\right). \end{array}$$

The indicators *δ*_*ct*_ have average 1 but are significantly lower than 1 for more outlying observations, such as daily spikes in cases.

Cross-validation estimations are made at points *M* < *T*. Thus, we consider twenty-day-ahead forecasts at three different stages of the UK COVID-19 epidemic. For the first cross validation, estimations are based on training data up to day *M*=80 with *F*=20 (i.e., the cross-validation period consists of days 81 to 100). Cross-validation estimations with *F*=20 are also made for *M*=100, and for *M*=120, with forecast accuracy based on comparing forecasts with hold-out data for days *M*+1,…, *M*+*F*.

### 4.2. Model Assumptions

For prior densities on the unknowns in the medium-term forecasts, exponential priors with mean 1 are assumed on *r*_*c*_, *r*_*d*_, *a*_*c*_, and *a*_*d*_. For the precisions 1/*σ*_*uc*_^2^ and 1/*σ*_*ud*_^2^ in the PLN and PLS options, and for the parameters *λ*_*c*_ and *λ*_*d*_ in the Poisson-gamma model, a gamma prior Gamma (1,0.001) is assumed. For the PLS option, we take *ν*_*c*_=*ν*_*d*_=4 as a preset option. The degrees of freedom can be difficult to estimate for relatively small datasets, and the option of the preset value *ν*=4 is a robust option [52, 53]. For the maximum cases (epidemic size) parameter *K*_*c*_, we assume as lognormal prior, centred at log(*K*_*c*_^*e*^) and with variance 1, as discussed in Section 3.2.

For the maximum deaths parameter *K*_*d*_, we assume *K*_*d*_=Φ*K*_*c*_, where the prior for Φ is beta distributed with mean defined by the EU-wide case fatality *ϕ*_EU_. The beta has total prior count *C* set at 5, *C*=5. Thus, the prior on Φ is Beta(*Cϕ*_EU_, *C*(1 − *ϕ*_EU_)). For example, on 20-04-2020 (at day *t*=80), the EU-wide case fatality rate was 0.101, and the prior is set at Φ ~ Beta (0.505, 4.495). This illustrates the use of relevant external information, rather than diffuse priors.

### 4.3. Model Fit and Estimation

Fit to the observed training data is assessed using the Watanabe–Akaike information criterion (WAIC) [54]. This is a fit measure which takes account of fit but also penalises model complexity. Cross-validation fit (fit out of the observed sample) is assessed by the probabilities of forecasting overprediction, *ω*_*c*_ and *ω*_*d*_, and by predictive coverage: whether the credible intervals for predicted cases and deaths (averages over each validation period) include the observed averages. Estimation uses the BUGS program [55], with posterior estimates based on the second halves of two chain runs of 100,000 iterations, and convergence assessed using Brooks–Gelman–Rubin criteria [56].

### 4.4. Modelling Later Epidemic Stages

To provide a longer-term perspective on epidemic containment, we apply the best performing model from analysis of the first wave to the full set of observations as at 8th August 2020 (*T* = 190), when the first epidemic peak had passed. So this application is to a situation where the epidemic first wave has passed, but there are still nonnegligible numbers of new cases, and a potential for possible upturns and further waves.

**Figure 3:**
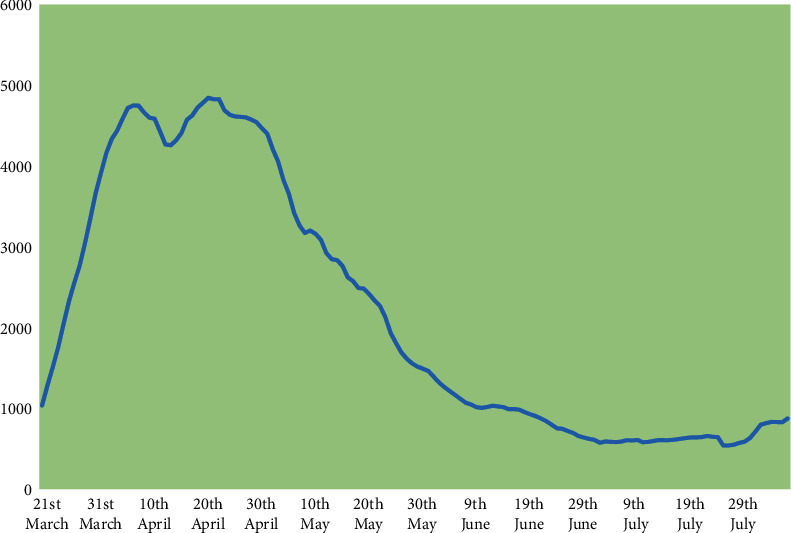
Seven-day moving average new cases, UK COVID epidemic.

New cases and deaths (as 7-day moving averages) had reached maxima of 4850 and 950, respectively, when the UK epidemic peaked in April 2020. As a result of lockdown measures imposed in late March, daily new cases averaged just over 500 daily by July. However, lockdown relaxations from July were accompanied by the risk of resurgence. In that regard, an upturn was apparent with new cases in late July and early August averaging over 800 daily (see Figure 3), although deaths continued to fall, averaging around 50 per day in early August.

To model the full time series, and since there was no upturn in deaths apparent in early August 2020, we focus on new cases only. To reflect the evidence of an upturn in cases, a two-phase model is applied. For this model, the shift parameter *κ*_*c*_ is assigned an exponential prior density with mean 150. Priors on the parameters of the second-phase Richards model are as before for the exponential ascent and logistic modifier parameters. For *K*_*c*2_ (the forecast total cases under wave 2), we assume *K*_*c*2_=*η*_*c*_*K*_*c*1_ with *η*_*c*_ assigned an exponential prior with mean 1. Of policy interest here is epidemic containment, as summarised by the effective reproduction ratio: specifically the question of policy relevance is whether this ratio consistently is below 1, and whether its 95% credible interval also is entirely below 1. If the ratio is not below 1, this suggests a significant upturn.

To provide estimates of the effective reproduction ratio, we use accumulated evidence on COVID serial intervals from five studies [57–61]. A gamma density on the serial interval (SI) is assumed, and information on mean and standard deviation of the SI, or on quantiles of SI, is converted to gamma density parameters; for use of SI quantiles in this regard, see [62]. Large samples (of a million) from each the five densities are taken, and parameters of the pooled gamma density are estimated from the pooled sample of five million; the pooled gamma density has shape 1.38 and rate 0.36. This density is then converted to a discretised form (with 16 bins) to provide an informative prior on the SI to the model of White and Pagano [63], which updates the prior serial interval density using new case data for the UK. The study in [64] recommends that the initial, approximately exponential, epidemic phase be used in estimation, and we use UK new case data up to time 24-04-2020, when UK new cases peaked (see 55, page 3). The updated mean serial interval is estimated as 3.5 days with standard deviation 3.1. The discretised serial interval is estimated as in [63], with *J*in Section 3.5 set at *J*=16.

## 5. Results

Table 1 compares parameter estimates from the observed (training) data under the three error assumptions for the three cross-validation analyses at *M* *=* 80, *M* = 100, and *M* = 120 (i.e., nine scenarios). It should be noted that predictions of cases and deaths should be based not on the posterior mean or median parameter values in Table 1, but on sampled posterior predictive replicate data at each iteration. These are based on sampling new data from the Poisson means (13) and (14), and from the Richards model parameter profile at each particular iteration. The predicted values of cases and deaths are very close to actual values: for example, for the PLS model at *M* = 120, the average absolute deviation (over *t*=2,…, 120) between actual new cases and predicted new cases is under 1.  Table 2 compares the WAIC fits to the observed data under the nine options, while criteria regarding 20-day-ahead forecasts are shown in Table 3.

### 5.1. Mid-Term Forecasts

Table 1 shows broad consistency between the three distributional options in terms of estimated final epidemic size *K*_*c*_ and eventual death total *K*_*d*_. The posterior density of these parameters may be skewed, with posterior mean exceeding median. For the PLN and PLS options, the estimate of *K*_*c*_ increases as *M* does. This reflects the protracted nature of the UK downturn in cases after the peak in the first wave. Posterior mean estimates of the turning points *τ*_*c*_ and *τ*_*d*_ vary slightly and tend to be higher for *M*=100 and *M*=120, but for both outcomes and all *M* values are between 72 and 86. Turning point estimates for new cases *τ*_*c*_ are also mostly higher under the PLS option.

Table 2 shows that the PLS option has better fit, with lower WAIC values. Hence, its estimates of epidemic size and turning points are preferred and provide a better description of the slow decline in cases from their peak. Table 3 shows generally better cross-validation performance for the PLS option. As discussed above, satisfactory prediction would have 0.05 < *ω*_*c*_ < 0.95, and 0.05 < *ω*_*d*_ < 0.95, with *ω*_*c*_ or *ω*_*d*_ over 0.95 indicating overprediction, and with *ω*_*c*_ or *ω*_*d*_under 0.05 indicating underprediction. Both PG and PLN options show underprediction for higher values of *M*, and in policy terms, may be misleading in suggesting a faster decline in cases and deaths than actually occurred. By contrast for *M*=100 and *M*=120, the 95% interval for predicted average cases under the PLS model comfortably includes the actual average new cases.

### 5.2. Longer-Term Scenario

Estimates of policy relevant parameters also come from the longer-term scenario when the log-Student Richards model is applied to UK COVID new case data up to day *T* = 190 (8th August, 2020). The shift point in the two-phase model is estimated as 179.1 with 95% interval from 179.0 to 179.3.

**Figure 4:**
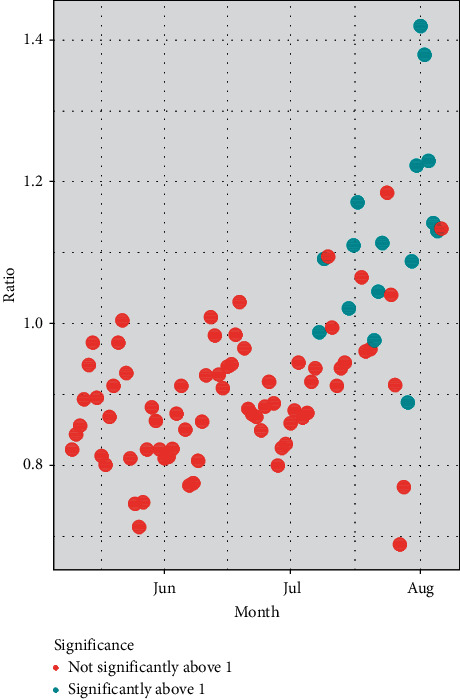
Reproduction ratios and significance.

Figure 4 plots out the posterior mean estimated effective reproductive ratios (5-day moving averages), distinguishing those estimates significantly above 1. The estimates hover around 1 throughout June and the first half of July but, in late July/early August, tend to exceed 1. The impression from this is that success in fully effective containment is then in doubt. The estimates of the reproduction ratio, and their path through June and July, are similar to those for the UK available at https://epiforecasts.io/covid/posts/national/united-kingdom/#national-summary and based on the methods in [65].

**Figure 5:**
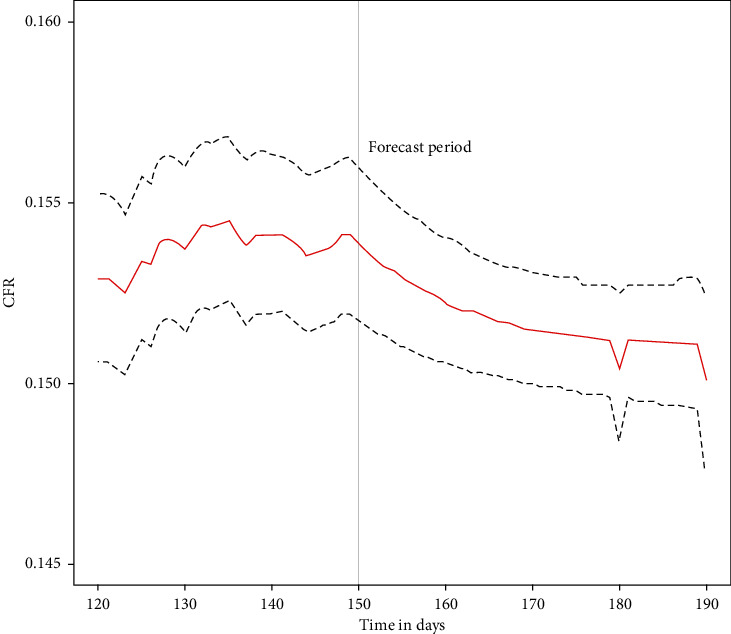
Modelled and forecast CFR.

To illustrate the potential for longer-term forecasts of severity indicators, we also use observed data up to day 150 to make forecasts 40 days ahead through to day 190. This analysis uses the PLS model option for Poisson mixing.  Figure 5 shows the resulting forecast of the case fatality ratio, with 95% intervals. The interval for *t*=190 includes the observed value of 0.151.

## 6. Discussion

The existing epidemic modelling literature has recognized the need for overdispersed distributions to deal with erratic incidence counts [29, 66, 67]. Thus, the study in [67] shows that use of a negative binomial distribution is more appropriate than the Poisson for describing emerging infections with overdispersed case distributions due to superspreading events. However, so far as the authors are aware, there has been no evaluation of the negative binomial as compared to other methods of representing overdispersion in epidemic counts. Hence, one contribution of this paper rests on a comparison of the negative binomial (Poisson-gamma mixture) against alternative Poisson mixture models [30]. For example, the Poisson log-normal distribution is a longer tailed alternative to the negative binomial distribution and may better fit overdispersed count data [68, 69]. The analysis here suggests such alternatives may usefully be considered as alternatives to the negative binomial and may both improve fit to actual observations and provide more accurate forecast performance.

Tackling overdispersion is one issue present in modelling count data associated with epidemics. Another is the outcome focus: the choice is between incidence alone (the usual approach), or on taking account of both incidence and related outcomes. We have developed here a bivariate approach to jointly modelling epidemic outcomes, using priors that link parameters between outcomes. For example, here, the eventual death total *K*_*d*_ is linked (via the Bayesian prior specification) to the eventual epidemic size *K*_*c*_. This approach has been used here to study the interrelationships between incidence and mortality but can be readily extended to more outcomes, such as incidence, mortality, and hospitalisations. Forecasts from such joint outcome models enable forecasts of epidemic severity (e.g., case fatality rates, case-hospitalisation ratios, deaths-hospitalisations ratios, and effective reproduction ratios) that assist in pandemic severity assessment. Severity assessment, as well as the development of summary indicators of epidemic severity, is being recognized as an important aspect of epidemic monitoring and modelling [70, 71].

The present paper also considers the gain in applying phenomenological models to later stages of incomplete epidemics, especially (in the case of COVID-19) after lockdown relaxations, where there may be a protracted period of nonzero incidence or further more pronounced waves of infection. The above analysis has therefore applied the Poisson-log-Student to late epidemic data for the UK (up to early August 2020), when there was some evidence of an incidence upturn, but not yet a full-blown second wave. The focus of this analysis was on the effective reproduction rate and the case fatality ratio, both indicators of epidemic severity. As mentioned in [72], “the COVID-19 pandemic has shown that the effective reproduction rate of the virus *R*_*t*_ is a crucial determinant not only of public health, but also of public policy.” There are a number of ways of estimating this quantity, including novel approaches such as using Google mobility data [73]. Here, an analysis using estimates of *R*_*t*_ based on a two-phase Richards model suggests an upturn in transmission in the UK by late July/early August 2020.

Hence, the added value of the study is provided by the following: (a) proposing a multivariate framework readily applied for assessing severity, (b) providing an approach for assessing alternative methods for representing overdispersion, and (c) providing a methodology for assessing long-term trends and making longer-term forecasts (e.g., of case fatality and the effective reproduction rate) in a multiwave situation.

## 7. Conclusion

Many applications of phenomenological models have been to complete epidemics. However, evaluation of such models based simply on their fit to observed data may give only a partial picture. Also relevant to epidemic model assessment, particularly for policy application, is the accuracy of medium-term forecasts for incomplete epidemics. Arguably, evaluation in this case is better done using a cross-validation approach, where only some of the observed data are used to estimate parameters, and a hold-out sample can then assess the accuracy of forecasts. The analysis here of UK epidemic data in the first half of 2020 has shown that fit to training data and the cross-validation fit are consistent in their choice of preferred model option.

The contribution of the present paper is to illustrate a bivariate approach to two epidemic outcomes, and how prior information (under a Bayesian approach) can be applied to interlink the parameters governing each outcome. The benefits of a bivariate (and potentially multivariate) approach include the ability to forecast severity measures such as the case fatality ratio, and the borrowing of strength over outcomes in making forecasts [26]. A further contribution is that a focus on incidence and new deaths rather than cumulative outcomes has brought into sharper focus the question of adequately representing Poisson overdispersion. The latter is caused by often erratic fluctuations in the observed series, apparent in UK COVID data on new cases and deaths. The analysis has provided new evidence on the relative fit and forecasting performance of different ways of representing Poisson overdispersion in epidemic count data, and potential gains through using heavier tail alternatives to the negative binomial. We have also considered Bayesian analysis of longer-term epidemic trends, where multiple waves may exist, and illustrated monitoring of case fatality and epidemic reproduction.

The implications of the research (e.g., in planning healthcare provision) are that effective medium-term forecasts of COVID-19 incidence and mortality can be provided by the proposed methodology. Extension of the bivariate approach (e.g., to include incidence, mortality, and hospitalisations) provides scope for forecasting other indicators relevant to severity assessment. The methodology also presents a way to monitor longer-term infection numbers leading to early detection of incipient upturns in infection numbers, via continuous monitoring and forecasting of the effective reproduction ratio. Assessing whether the latter is confined to values below 1 is important for strategic epidemic containment.

The present study has some possible limitations. Comparative analysis of alternative ways of representing overdispersion has been limited here to UK data, and to the first wave COVID-19 epidemic, and will require validation with other epidemic time series, both for COVID-19 and other infectious diseases. A caveat, although not a limitation per se, is that, with regard to Bayesian estimation, relatively informative priors may be needed to guarantee stable estimation and ensure convergence. For example, diffuse gamma priors on the eventual epidemic size parameter caused convergence problems in the analysis reported here.

Regarding future research, as just pointed out, the methodology for comparing overdispersion approaches should be assessed with other epidemic datasets. Other Poisson mixtures may be considered such as the Poisson log skew-normal [74] or Poisson mixtures with other densities [75, 76]. Other types of analysis regarding forecasting potential can also be envisaged, such as forecast combinations, for instance, forecasts based on combining the logistic, Richards, and Gompertz curves [77, 78]. It would also be useful, especially in planning hospital care capacity, to apply a bivariate approach to COVID-19 incidence and hospitalisations, or a trivariate approach to incidence, mortality, and hospitalisations. A trivariate approach will provide model estimates and forecasts of indicators central to severity assessment.

## Figures and Tables

**Table 1:** Estimations according to timing of cross-validation period (*M* is days after start of epidemic).

Parameter	Poisson-gamma				Poisson-lognormal				Poisson-log-Student			
	Mean	2.5%	Median	97.5%	Mean	2.5%	Median	97.5%	Mean	2.5%	Median	97.5%
*M* = 80												
**K**_**c**_	373800	151800	210500	1172000	170200	135900	153200	289000	198800	135100	177200	336600
**K**_**d**_	32920	25070	32080	45560	33220	24180	32390	46770	35780	17950	29060	91460
**r**_**c**_	0.31	0.22	0.27	0.58	0.24	0.19	0.23	0.30	0.12	0.08	0.13	0.18
**r**_**d**_	0.82	0.44	0.75	1.51	0.80	0.38	0.67	1.81	0.29	0.17	0.28	0.46
**a**_**c**_	0.31	0.07	0.32	0.60	0.54	0.21	0.55	0.89	1.06	0.53	1.06	1.79
**a**_**d**_	0.13	0.05	0.12	0.26	0.14	0.04	0.13	0.33	0.75	0.20	0.63	1.75
**Φ**	0.13	0.03	0.15	0.25	0.21	0.10	0.20	0.31	0.18	0.09	0.16	0.40
***τ***_**c**_	76.2	72	75	80	72.5	70.0	72.0	80.0	76.1	73.0	75.0	80.0
**τ**_**d**_	74.5	71	74	79	74.7	71.0	75.0	80.0	75.8	71.0	76.0	80.0

*M* = 100												
**K**_**c**_	271040	243490	268820	310190	262700	245700	261100	284000	295800	223800	276400	453200
**K**_**d**_	37395	35097	37274	40538	37040	34470	36920	40340	38740	32290	37610	53060
**r**_**c**_	0.30	0.22	0.30	0.38	0.26	0.22	0.26	0.33	0.18	0.13	0.17	0.27
**r**_**d**_	1.51	0.63	1.21	4.01	1.27	0.48	1.06	2.88	0.55	0.22	0.49	1.12
**a**_**c**_	0.25	0.17	0.24	0.42	0.28	0.20	0.28	0.37	0.46	0.20	0.46	0.83
**a**_**d**_	0.07	0.02	0.06	0.13	0.08	0.02	0.07	0.17	0.20	0.06	0.15	0.62
**Φ**	0.14	0.12	0.14	0.16	0.14	0.13	0.14	0.16	0.14	0.08	0.13	0.20
***τ***_**c**_	79.4	78	79	82	79.0	78	79	80	83.1	78	82	97
**τ**_**d**_	76.2	75	76	77	76.1	75	76	77	77.6	75	77	83

*M* = 120												
**K**_**c**_	302800	295900	302300	312100	303100	293900	302700	317100	349100	282000	321300	549100
**K**_**d**_	40670	39670	40630	41890	40370	39410	40330	41560	41050	38580	40770	45020
**r**_**c**_	0.38	0.30	0.36	0.51	0.34	0.25	0.32	0.49	0.40	0.11	0.45	0.80
**r**_**d**_	1.87	0.65	1.50	5.38	1.57	0.67	1.52	2.82	0.94	0.34	0.78	2.35
**a**_**c**_	0.16	0.11	0.16	0.21	0.18	0.11	0.18	0.24	0.27	0.04	0.30	0.69
**a**_**d**_	0.05	0.01	0.04	0.11	0.05	0.02	0.04	0.10	0.09	0.03	0.08	0.22
**Φ**	0.13	0.13	0.13	0.14	0.13	0.13	0.13	0.14	0.12	0.07	0.13	0.15
***τ***_**c**_	81.0	80	81	82	81.2	81	81	82	85.6	82	84	99
**τ**_**d**_	77.3	77	77	78	77.1	77	77	78	77.9	77	78	79

**Table 2:** Fit to training data (WAIC criterion).

	Poisson-gamma	Poisson-lognormal	Poisson-log-Student
*M* = 80			
Cases	605.0	600.0	564.1
Deaths	365.7	367.4	350.4
Total	970.7	967.4	914.5

*M* = 100			
Cases	851.6	845.8	808.8
Deaths	562.0	560.1	550.1
Total	1413.6	1405.9	1358.9

*M* = 120			
Cases	1097.0	1086.3	1042.3
Deaths	743.3	741.4	731.5
Total	1840.4	1827.7	1773.8

**Table 3:** Criteria for out-sample predictions (*M* is number of days in training sample; *F* = 20 in all cases).

Criterion	Poisson-gamma (PG)				Poisson-lognormal (PLN)				Poisson-log-Student (PLS)			
	Mean	2.5%	Median	97.5%	Mean	2.5%	Median	97.5%	Mean	2.5%	Median	97.5%
*M* = 80												
Average daily cases	4357	1818	3686	7407	2214	1078	1881	4875	3118	1043	2869	5652
Average daily deaths	609	344	603	904	613	303.8	613.4	921.8	587	0	514	1471
*ω*_*c*_	0.41				0.03				0.16			
*ω*_*d*_	0.34				0.38				0.40			
Actual daily averages in period (*M* + 1, *M* + *F*)												
New cases	4857											
New deaths	662											

*M* = 100												
Average daily cases	2346	1523	2353	3173	1975	1384	1947	2658	2630	622	2565	4340
Average daily deaths	265	184	265	351	224.8	135.8	221.5	332.8	275	52	276	510
*ω*_*c*_	0.09				0.00				0.31			
*ω*_*d*_	0.06				0.03				0.32			
Actual averages in period (*M* + 1, *M* + *F*)												
Cases	3039											
Deaths	330											

*M* = 120												
Average daily cases	1132	1006	1129	1274	1133	963.2	1136	1320	1310	604	1358	1835
Average daily deaths	129	89	129	170	117.9	77.6	117.6	160.4	135	37	134	236
*ω*_*c*_	0.00				0.00				0.42			
*ω*_*d*_	0.00				0.00				0.07			
Actual averages in period (*M* + 1, *M* + *F*)												
Cases	1506											
Deaths	216											

## Data Availability

The data used to support the findings of the study were obtained from the European Centre for Disease Prevention and Control (https://www.ecdc.europa.eu/en/publications-data/download-todays-data-geographic-distribution-covid-19-cases-worldwide).
